# An Investigative Study on the Oral Health Condition of Individuals Undergoing 3D-Printed Customized Dental Implantation

**DOI:** 10.3390/jfb15060156

**Published:** 2024-06-05

**Authors:** Hongyang Ma, Yuqian Kou, Hongcheng Hu, Yuwei Wu, Zhihui Tang

**Affiliations:** The Second Dental Center, Peking University School and Hospital of Stomatology, Beijing 100101, China; mahongyang1991@foxmail.com (H.M.);

**Keywords:** 3D printing, additive manufacturing, custom dental implant, osseointegration, zirconia

## Abstract

Background: The advent of three-dimensional (3D) printing technology has revolutionized the field of dentistry, enabling the precise fabrication of dental implants. By utilizing 3D printing, dentists can devise implant plans prior to surgery and accurately translate them into clinical procedures, thereby eliminating the need for multiple surgical procedures, reducing surgical discomfort, and enhancing surgical efficiency. Furthermore, the utilization of digital 3D-printed implant guides facilitates immediate restoration by precisely translating preoperative implant design plans, enabling the preparation of temporary restorations preoperatively. Methods: This comprehensive study aimed to assess the postoperative oral health status of patients receiving personalized 3D-printed implants and investigate the advantages and disadvantages between the 3D-printed implant and conventional protocol. Additionally, variance analysis was employed to delve into the correlation between periodontal status and overall oral health. Comparisons of continuous paired parameters were made by *t*-test. Results: The results of our study indicate a commendable one-year survival rate of over 94% for 3D-printed implants. This finding was corroborated by periodontal examinations and follow-up surveys using the Oral Health Impact Profile-14 (OHIP-14) questionnaire, revealing excellent postoperative oral health status among patients. Notably, OHIP-14 scores were significantly higher in patients with suboptimal periodontal health, suggesting a strong link between periodontal health and overall oral well-being. Moreover, we found that the operating time (14.41 ± 4.64 min) was less statistically significant than for the control group (31.76 ± 6.83 min). Conclusion: In conclusion, personalized 3D-printed implant surgery has emerged as a reliable clinical option, offering a viable alternative to traditional implant methods. However, it is imperative to gather further evidence-based medical support through extended follow-up studies to validate its long-term efficacy and safety.

## 1. Introduction

Dental implants have become a popular and successful solution for tooth replacement, offering patients a permanent and natural-looking restoration option since Prof. P.I. In 1965, Brandmark successfully implanted titanium implants into a patient’s mandible [1]. In 1998, German dentist Dr. Paul Weigl successfully utilized computer-aided design and manufacturing (CAD/CAM) technology to produce dental implants and their attachments. Since then, dental implantology has undergone continuous development and innovation [2]. Modern dental implantology utilizes advanced materials, imaging technology, and surgical techniques to provide patients with safer, more reliable, and aesthetically pleasing solutions for tooth replacement [3,4]. 3D printing, also referred to as additive manufacturing, has brought significant advancements to various industries, including the fields of healthcare and dentistry [5]. In recent years, the application of 3D printing technology in dental implantology has gained momentum, allowing the production of customized and highly accurate implant components [6]. 

Traditional methods of manufacturing dental implants often involve manual labor and may not consistently achieve an optimal fit and aesthetics. In contrast, 3D printing enables dental professionals to design and fabricate implant components such as abutments, crowns, and surgical guides with unparalleled precision and efficiency [7]. By utilizing digital scans of the patient’s oral cavity, image analysis based on cone beam computed tomography (CBCT), and personalized dental implant design, 3D printing technology enables the creation of tailored implants that perfectly adapt to individual anatomical structures [8].

The placement of dental implants depends on various factors, including anatomical conditions such as adequate bone height and thickness [9]. Consequently, personalized dental implants appear to be advantageous for patients with alveolar bone resorption [10]. Additive manufacturing technology has facilitated the production of customized implants with precise microscale resolutions [11]. Ti-6Al-4V powder is processed into cylindrical substrates for surface heat treatment. Subsequently, half of these substrates are annealed under an argon atmosphere and then cooled down. Additionally, Ti-6Al-4V powder, through stereolithography (SLA), is molded for sandblasting and acid erosion procedures. Half of these squares undergo sandblasting with pure titanium powder [12]. The integration of 3D-printed dental implants presents several advantages including enhanced accuracy, reduced production time, and improved patient outcomes. Furthermore, 3D printing allows for the cost-effective production and customization of dental implants, making them more accessible to a broader population [13].

Despite animal studies and individual case reports regarding personalized 3D-printed dental implants, there is a scarcity of prospective cohort studies with long-term follow-up to provide substantiated evidence [14]. Therefore, this study aimed to assess oral health outcomes following personalized 3D-printed implant procedures by conducting comprehensive clinical examinations, including periodontal assessments, oral health evaluations, and auxiliary examinations such as intraoral photography, X-rays, and CBCT. 

## 2. Materials and Methods

Patients who underwent personalized 3D-printed dental implant surgery between January 2021 and March 2023 were recruited for this study. At the same time, routine immediate dental implant patients’ data were compared with 3D-printed implant (experimental group) data as a control group. The experiments conducted on human participants in this study adhered to the ethical guidelines set by Peking University, Beijing, China (approved by the Biomedical Ethics Committees of Peking University School and the Hospital of Stomatology, with the approval number PKUSSIRB-201734034) and complied with the principles outlined in the 1964 Declaration of Helsinki and subsequent revisions or similar ethical standards. The study inclusion criteria were individuals aged 18–70 years, with no restrictions on sex; patients requiring immediate implantation after tooth extraction for clinical diagnosis; a root length of the dental bone segment of ≥7 mm; intact buccal and lingual alveolar bone, with a bone plate thickness ≥1.5 mm; a width of the keratinized gingiva on the buccal side exceeding 2 mm; a thick gingival biotype; presence of adjacent normal natural teeth; absence of obvious inflammation or injury in the oral mucosa and soft tissues; the condition of the remaining dentition, edentulous spaces, occlusal relationship, jawbone morphology, and mouth opening meeting the requirements for restoration; ability to communicate effectively with the researchers; good compliance; and voluntary willingness to adhere to the follow-up requirements according to the trial protocol. The exclusion criteria were the following: excessively high aesthetic expectations; metal allergies; fear of radiation; patients with acute or specific infections (including STDs, tuberculosis, etc.); patients with primary diseases of the heart, brain, liver, kidney, hematopoietic system, endocrine system, and mental illness; patients with severe diabetes (fasting blood glucose ≥ 9 mmol/L or glycosylated hemoglobin exceeding 10%); patients with a history of head and neck radiotherapy or chemotherapy within 5 years; patients with metal allergies or allergies to related drugs; patients taking steroids or other medications that may have affected postoperative healing or osseointegration; patients with alcohol dependence, drug addiction, or a tendency toward addiction; smoking more than 10 cigarettes per day; pregnant or lactating women; patients with jaw fractures; patients with severe habitual teeth grinding; patients with benign/malignant tumors in the oral cavity or jawbone; patients in the surgical area adjacent to teeth that had undergone root canal treatment or had periapical cysts; and patients with poor compliance.

**Figure 1:**
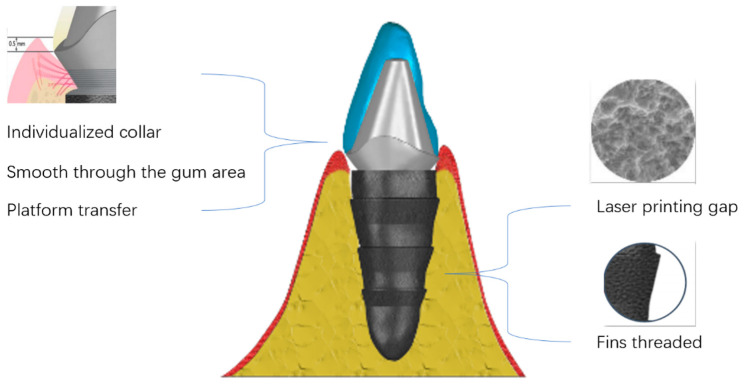
Mechanistic diagram of 3D-printed implants.

**Figure 2:**
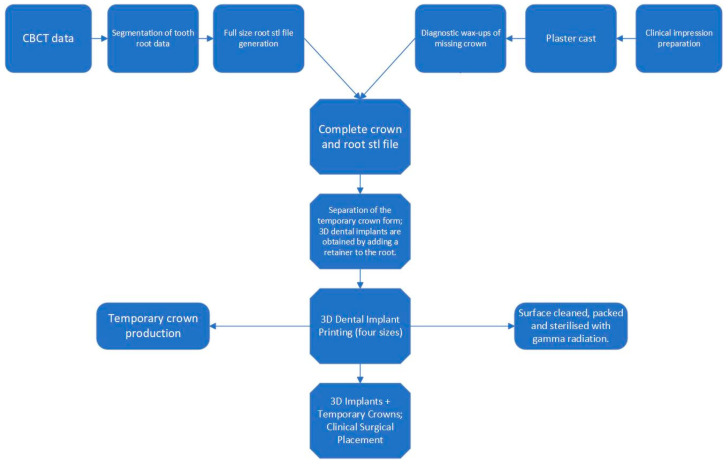
Flowchart of the fabrication of 3D-printed personalized oral implants.

Reconstruction of three-dimensional models of the alveolar sockets and teeth was achieved using Mimics Research 19.0 (Materialise, Leuven, Belgium); then, the reconstructed models were imported and saved as STL files. All STL datasets were imported into Geomagic Studio 12 (Geomagic, Morrisville, NC, USA) to design individualized dental implants. 3D-printed implants were processed in the following steps: Ti-6Al-4V powder was processed into cylindrical substrates for surface heat treatment. Subsequently, half of these substrates were annealed at 820 °C for 4 h under an argon atmosphere and then cooled down. Additionally, Ti-6Al-4V powder, through stereolithography (SLA), was molded into 15 mm × 15 mm squares with a thickness of 1 mm for the sandblasting and acid erosion procedures. Half of these squares underwent sandblasting with 25–100 μm pure titanium powder. Following this, they were treated with a 10% HCl/H_2_SO_4_ mixture at a temperature of 100 °C or above for 3 min, culminating in a thorough cleaning process. Personalized 3D-printed oral implants in 90%, 95%, 100%, 105%, and 110% sizes were printed using an electron beam melting 3D printer (ARCAM Q10plus; Mölndal, Sweden) prepared prior to surgery. The mechanism diagram of 3D-printed implants is illuminated by Figure 1. Clinical photography captured information on tooth roots and crown morphology. CBCT scanning segment data were converted to STL files. A temporary crown was shaped based on crown requirements, then a 3D dental implant (abutment-integrated) was created. STL data were sent to the laboratory for fabrication. Finally, the customized dental implant underwent cleaning, sterilization, polishing, and fitting. 3D implants were implanted from the smallest size and replaced with larger sizes until stable (Figure 2). 

During this procedure, the unreserved tooth was extracted using minimally invasive instruments, and the implant was inserted into the socket by the operator using the impact method, which uses the best matching size of an implant depending on the stability of the implant.

**Figure 3:**
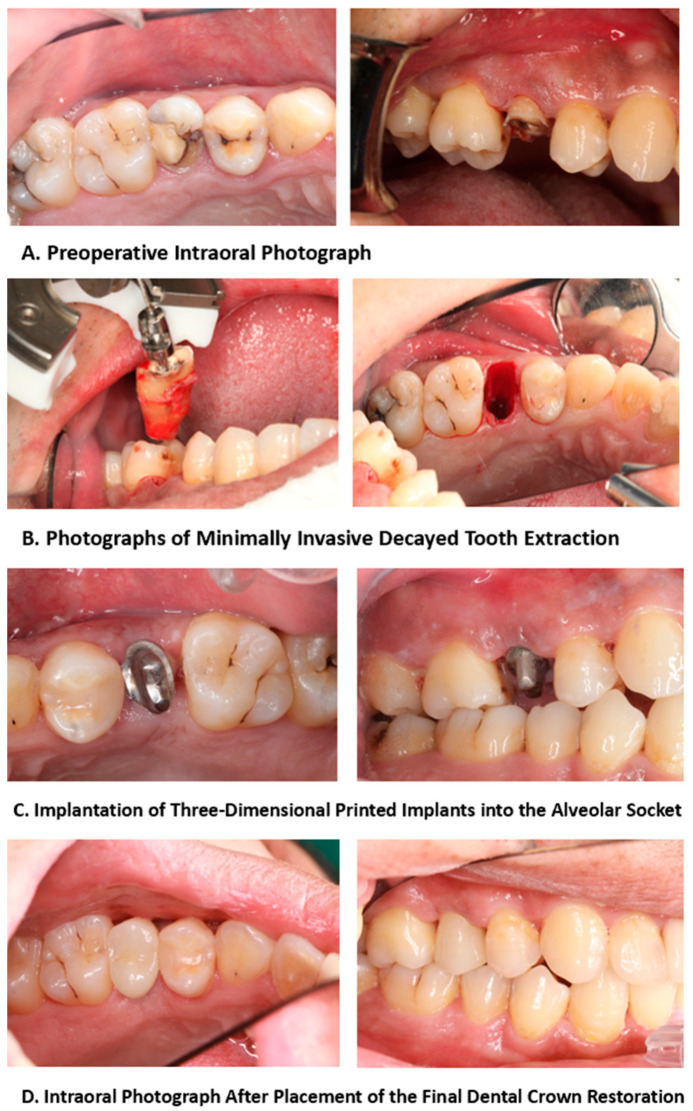
Treatment and follow-up process of 3D-printed implants.

**Figure 4:**
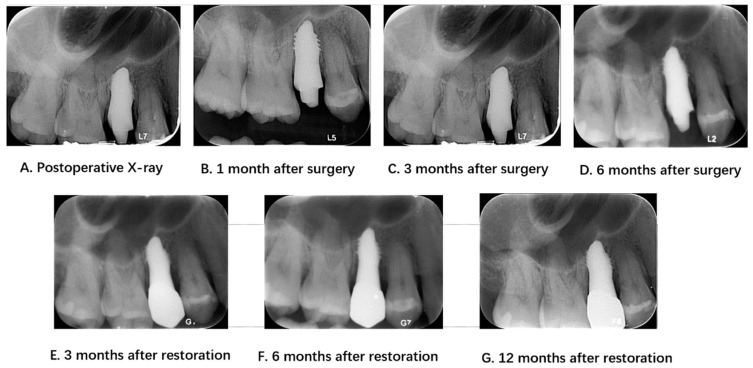
Radiological examinations before and after surgery and during the follow-up period.

During the follow-up period, patients underwent a preoperative oral examination, which included classifying and assessing the bone quality of the pre-implantation site; the reasons for tooth extraction; the degree of looseness of the teeth to be extracted; the condition of the jaw teeth; the occlusal status; and the status of adjacent teeth in the anterior, posterior, and middle regions. The treatment and follow-up workflow of the 3D-printed implants is summarized in Figure 3. In addition, patients underwent periodontal examinations, including assessments of plaque accumulation, calculus index, periodontitis, buccal attached gingiva, gingival biotype, and whether the periodontal condition met the implantation requirements. Radiographic examination results included a description of the tooth root conditions, the length of the dental bone segment within the root, and the thickness of the buccal and lingual alveolar bone (Figure 4). The Oral Health Impact Profile (OHIP-14) questionnaire was used to investigate the oral health status of patients after oral implant surgery for at least 12 months. 

The mean was reported for the continuous parameters. The normally distributed continuous data were compared by one-way analysis of variance (ANOVA) (SPSS, IBM SPSS 23.0; SPSS Inc., Chicago, IL, USA). Comparisons of continuous paired parameters between the experimental group and control group were made by *t*-test. If the data were not normally distributed, the Wilcoxon rank sum test and *t*-test were used. A *p*-value of <0.05 was considered statistically significant. 

## 3. Outcomes

Seventeen patients (five male and twelve female) were included in this study. The mean age of the patients was 39 years (range, 28–62 years), and the mean follow-up time was 25 months (range, 12–36 months). All participants in this study experienced unretained teeth as a result of caries, pulpitis, and dental trauma. The post-implant restorative protocol was a restoration with zirconia for at least three months after dental implant placement. All patients had satisfactory outcomes without major complications, except for one case with implant failure due to unfavorable osseointegration (Table 1). 

The dental implant success criteria were the following: clinical examination of the implant without loosening; no translucent area around the implant in X-ray examination; vertical bone resorption less than 1.5 mm in the first year after implant placement, and less than 0.2 mm in the subsequent annual review of vertical bone resorption; no persistent and/or irreversible symptoms and signs after implantation, such as pain, infection, hypersensitivity, sensory abnormalities, and neural tube damage; and satisfactory aesthetic effect after restoration [15]. The one-year follow-up of patients who received 3D-printed implants, assessed through periodontal examinations and OHIP-14 questionnaire follow-up, revealed a high survival rate of over 94%. 

**Figure 5:**
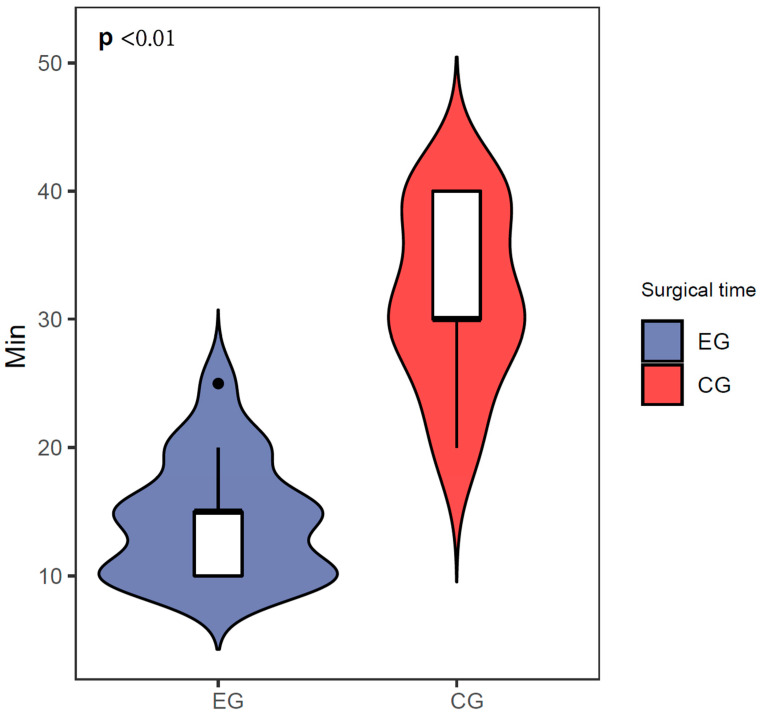
Surgical time comparison between the experimental group and control group.

Patients also exhibited excellent postoperative oral health status. The results of the OHIP-14 oral health questionnaire (Table 2) showed that seven patients who received dental implants believed that their dietary habits would be somewhat affected. Six patients reported that they would pause during meals and could not eat continuously. After receiving the implants, there was little to no impact on patients’ pronunciation or daily activities. Furthermore, the survey also revealed that patients with periodontitis had lower scores than those with healthy periodontal conditions (*p* < 0.01), indicating that periodontitis can have a negative impact on the oral health of dental implant patients. Patient data for the control group are shown in Table 3. In contrast with the control group, we found that the operating time (14.41 ± 4.64 min) was less statistically significant than for the control group (31.76 ± 6.83 min, Figure 5).

## 4. Discussion

Through a retrieval of the literature, we found that this study is one of the longest available studies on the application of 3D-printed dental implants to clinical samples with a follow-up. The first treatment steps were significantly simplified; eight steps for the traditional machined finished products (minimally invasive extraction, tooth implant preparation, covering, bone increment, buried healing, second-stage surgery, abutment, temporary crown and permanent crown installation) were simplified to three steps (minimally invasive extraction, implant implementation, crown restoration), which reduced the number of patient visits and length of medical visits. Moreover, the second treatment cycle was significantly shortened from 3 to 8 months of conventional planting to 4–12 weeks. Additionally, the operating time was reduced such that doctors could serve more patients. Surgical techniques were simplified and reduced from difficult to simple, with the 80-h training of physicians routinely required reduced to 8 h. Finally, the cost was reduced by half, from the traditional CNY 16,000 to CNY 8000, effectively reducing the patient’s financial burden. However, personalized root-shaped dental implants had a narrow range of adaptations and were generally not suitable for multi-root molars. Furthermore, the increased preoperative planning time and labor costs cannot be ignored (Table 4). 

The published research also demonstrated in part the effectiveness of our clinical research and revealed a scarcity of long-term follow-up studies that have specifically examined the outcomes of 3D-printed dental implants. Thaisa et al. concluded that additive manufacturing, a novel technology, had the potential to address numerous challenges across various disciplines, by performing a systematic review. However, in the field of dentistry, additional research was required to enhance the manufacturing process for custom dental implants, as a standardized methodology was lacking. Furthermore, the advantages and disadvantages associated with this technique had yet to be definitively defined [16].

The present study stands out as it has the largest sample size and the longest duration of prospective follow-up among all previous investigations. This study marks the first instance of employing highly accurate personalized 3D-printed implants in human subjects. These results unequivocally validated the elevated success rate associated with 3D-printed dental implants. Moreover, the implantation procedure requires less surgical time than traditional approaches which involve socket preparation. As part of the treatment protocol, patients are fitted with aesthetically pleasing personalized temporary crowns immediately prior to surgery. Notably, these crowns are designed to ensure minimal occlusal contact, which prevents occlusal forces from being exerted. Consequently, esthetic complications arising from the absence of a natural tooth during the interim phase can be effectively avoided. Following the establishment of osseointegration between the implant and alveolar socket, the patient proceeded to undergo trial fitting of the final restorative crown. Importantly, the 3D-printed dental implants employed in this study are meticulously engineered as integrated implant abutment systems, guaranteeing robustness and mitigating risks, such as abutment loosening or screw fracture. The implant geometry can be influenced by isolated parameters (thread type, thread pitch, thread depth, and face angle). Normally, implant surfaces are characterized by uniform threading, which not only facilitates optimal bone integration but also provides desirable initial stability due to their balanced compressive and stress and promise of a minimal shear force [17,18].

Personalized 3D-printed implants have many advantages over conventional implants in terms of processing and fabrication. In the penetrating gingival area, the personalized shoulder table is located 0.5 mm below the gums, which facilitates cleaning. The smooth surface makes plaque less likely to remain. The laser-etched texture prevents migration of the binding epithelium’s root side. The platform transfer favors the maintenance of the alveolar bone height and maintains the biological width. In the embedded part of the alveolar bone, selective laser melting treats the surface of the implant to form a primary void, followed by sandblasting and acid washing to form a secondary void that facilitates osteoclast adhesion. The design of the fin thread facilitates the initial stability of the implant as well as occlusal force transmission. The computer-aided design of the rooted 3D-printed implant ensures precise implant placement and maximum bone preservation [19]. Setting different sizes of personalized 3D-printed oral implants (90%, 95%, 100%, 105%, 110%) ensures that the implants can achieve a better initial stability, which is of great significance for the success of the implant surgery, and we found that most of the patients in our practice used the 105% size of the implants. This is because the size of the implant is consistent with the principle of bone extrusion into the alveolar fossa formed by traditional step-by-step reaming, and when the implant size slightly exceeds the alveolar fossa, it can form a better initial stability, which is more favorable for the integration of the implant and the alveolar bone. In addition, this procedure effectively reduces the possibility of bone burns owing to the passive step-by-step process [20]. As no additional gingival incision is required, the patient is less traumatized and the postoperative reaction is less severe [21]. The average operating time is approximately 10 min, which is considerably shorter than that of conventional implant surgery, and the number of patient visits was reduced because the crowns were designed before the operation [22]. Furthermore, 3D-printed implant surgery is inexpensive compared to traditional implant surgery and is not overly expensive [23]. Therefore, it is more universal and can meet the needs of all people.

In this study, a standardized OHIP-14 questionnaire was used to assess oral health. This questionnaire consists of 14 items, each measured on a five-point Likert scale ranging from “very often” to “never” [24]. By scoring patients’ responses, the evaluation system aims to achieve a more objective assessment, minimizing subjective judgments from both medical professionals and patients. Most patients attained favorable scores on the oral health scale, although average feedback was received for the assessment of dietary habits and difficulties during eating. This could be attributed to the patients’ cautiousness in exerting force despite receiving dental implants and obtaining dentures. Furthermore, analysis of periodontal condition records revealed that patients with periodontitis scored lower on oral health evaluation than those with healthy periodontal conditions. This discrepancy can be ascribed to oral health issues associated with periodontitis, including gingival bleeding, attachment loss, alveolar bone resorption, gingival recession, and tooth loosening. Notably, in one case, implant loosening occurred because of inadequate osseointegration, necessitating implant removal during the follow-up period. Traditional implantation was subsequently recommended as a remedial measure. When examining the factors contributing to implant failure, it is possible that patients with poor bone structure or inflammation in the apical area may experience bone resorption. Additionally, inadequate matching between the contour of the designed personalized implant and alveolar socket during the design process, as well as trauma to the alveolar socket during tooth extraction due to operative reasons, may have played a role in the failures. Another possible factor is the early biting of hard substances, which could have led to premature loading of the implant, resulting in implant failure. Therefore, it is particularly important to assess the risk of each case as it is the key to the long-term retention of dental implants. For junior dental implant specialists, the Straightforward, Advanced, Complex (SAC) assessment tool can be an effective way to assess the risks of implant surgery [25].

A limitation of this study is the absence of a double-blind randomized controlled trial and the lack of a comparative study with traditional surgery as the control group, which prevented conclusive evidence from being obtained favoring personalized 3D-printed implants over traditional implants. This study focused solely on single-rooted tooth implantation and did not include inclusion criteria for multi-rooted teeth, such as molars, which limits its clinical applicability. Furthermore, there was a lack of analysis regarding the extent of peri-implant alveolar bone resorption and the bite force.

## 5. Conclusions

After one year of clinical follow-up, the 3D-printed implants demonstrated a high survival rate, and the patients met all oral implant criteria. Poor periodontal condition was associated with lower oral health assessment questionnaire scores. Long-term follow-up studies are required to gather evidence for evidence-based medicine.

## Figures and Tables

**Table 1:** 3D-printed implant patient characteristics.

No.	Gender	Age (Y)	Tooth Position	Etiology	Root Length (mm)	Mesiodistal Diameter (mm)	Buccolingual Diameter (mm)
1	M	31	15	Residual Crown	9.5	3.5	8.8
2	F	33	35	Tooth Split	9.7	3.5	5.5
3	F	37	45	Residual Root	10.9	3.8	5.9
4	F	29	25	Residual Crown	11.3	4	7.3
5	F	33	24	Residual Crown	8.0	3.7	7.4
6	F	30	15	Residual Root	8.6	3.4	7.7
7	F	50	12	Residual Root	7.9	4.4	5.7
8	F	56	25	Residual Root	8.5	3.4	7.8
9	M	39	25	Residual Root	9.5	3.8	8.5
10	F	62	45	Residual Crown	7.8	4.0	5.9
11	F	23	15	Residual Crown	12.0	6.3	13.0
12	F	51	22	Residual Crown	9.9	4.0	5.8
13	F	30	15	Residual Crown	10.6	4.2	7.9
14	M	39	35	Residual Root	10.5	4.1	6.4
15	M	38	25	Tooth Split	13.5	4.5	9.1
16	M	28	35	Residual Root	11.7	5.0	6.9
17	F	37	15	Residual Crown	9.0	4.3	8.2

**Table 2:** OHIP-14 scale analysis of 3D-printed implant patients.

P No./OHIP No.	1	2	3	4	5	6	7	8	9	10	11	12	13	14	Total P	Periodontal Condition	Surgery Time (min)
1	0	0	0	0	0	0	0	0	1	1	0	0	1	0	3	0	15
2	1	0	0	0	0	0	0	0	0	0	0	0	0	0	1	0	10
3	0	0	0	0	1	0	0	0	0	0	0	0	0	1	2	0	15
4	0	0	0	0	0	0	2	0	0	1	0	2	1	0	6	0	10
5	0	0	0	0	0	0	0	0	0	0	0	0	0	0	0	0	20
6	0	0	1	0	0	0	2	0	0	2	0	0	0	0	5	0	20
7	0	0	0	0	0	0	0	0	0	0	0	0	0	0	0	0	10
8	0	0	0	0	0	1	0	0	0	2	0	0	0	0	3	0	15
9	0	0	1	0	0	0	3	2	0	0	0	0	0	0	6	0	10
10	1	1	0	4	1	1	4	4	0	1	1	1	1	0	20	1	15
11	0	0	0	0	0	0	0	0	0	0	0	0	0	0	0	0	15
12	0	2	2	2	1	2	3	2	0	1	2	2	1	0	20	1	10
13	0	2	0	0	0	2	3	4	0	0	0	2	2	0	15	1	15
14	0	0	2	3	1	0	1	2	2	0	2	1	0	0	14	1	25
15	0	0	0	0	0	0	0	1	0	0	0	0	0	0	1	0	10
16	0	0	0	0	0	0	0	0	0	0	0	0	0	0	0	0	20
17	0	0	0	0	0	0	0	0	0	0	0	0	0	0	0	0	10
Total OHIP	2	5	6	9	4	6	18	15	3	8	5	8	6	1			Average: 14.4

P No.: patient number; OHIP No.: Oral Health Impact Profile-14 number; Periodontal Condition 0: periodontal disease effectively controlled; Periodontal Condition 1: periodontal disease not effectively controlled.

**Table 3:** Patient characteristics in the controlled group.

No.\Parameter	Gender	Age (Y)	FDI	Implant Brand	Implant Size (mm)	GBR	Surgical Time
1	F	37	23	Straumann	3.3 × 10	N	30
2	M	60	14	Astra	3.5 × 8	Y	40
3	F	44	14	Straumann	4.1 × 10	N	25
4	F	64	45	Astra	3.5 × 9	N	30
5	M	60	24	Astra	3.5 × 9	N	30
6	F	50	11	Straumann	3.3 × 12	Y	35
7	F	25	21	Straumann	3.5 × 11	Y	35
8	M	70	24	Astra	4.0 × 8	Y	30
9	F	49	22	Straumann	3.3 × 10	Y	40
10	M	49	14	Straumann	4.1 × 10	N	20
11	M	35	21	Straumann	3.3 × 10	Y	40
12	F	49	15	Astra	4.0 × 11	N	20
13	F	32	14	Astra	3.5 × 9	N	25
14	F	48	15	Straumann	4.1 × 10	N	30
15	F	42	21	Straumann	3.3 × 12	Y	40
16	F	27	15	Straumann	4.1 × 10	N	30
17	F	44	21	Straumann	3.3 × 10	Y	40

FDI: World Dental Federation; GBR: guided bone regeneration; F: female; M: male; Y: yes; N: no.

**Table 4:** Comparison of advantages and disadvantages between conventional dental implants and 3D-printed dental implants.

	Conventional Dental Implants	3D-Printed Implants
Cost	16,000 RMB	8000 RMB
Surgical time	20–40 min	10–20 min
Planning time	5–10 min	60–120 min
Bone graft	Often	Less
Bone loss	Yes	No
Complication	Less	Less
Number of visits	3–4 times	2 times
Treatment steps	Normal	Simplified
Surgical technical difficulty	Normal	Simplified
Scope of application	Wide	Narrow

The cells marked as green represent an optimized outcome.

## Data Availability

The datasets generated and/or analyzed during the current study are available from the corresponding author upon reasonable request, subject to the permission of the institutional review boards of the participating institutions.
